# Membrane Proteomics Analysis of the *Candida glabrata* Response to 5-Flucytosine: Unveiling the Role and Regulation of the Drug Efflux Transporters CgFlr1 and CgFlr2

**DOI:** 10.3389/fmicb.2016.02045

**Published:** 2016-12-21

**Authors:** Pedro Pais, Carla Pires, Catarina Costa, Michiyo Okamoto, Hiroji Chibana, Miguel C. Teixeira

**Affiliations:** ^1^Department of Bioengineering, Instituto Superior Técnico, Universidade de LisboaLisbon, Portugal; ^2^Biological Sciences Research Group, Institute for Bioengineering and Biosciences, Instituto Superior TécnicoLisboa, Portugal; ^3^Medical Mycology Research Center, Chiba UniversityChiba, Japan

**Keywords:** antifungal drug resistance, flucytosine, CgFlr1 and CgFlr2, *Candida glabrata*, CgPdr1

## Abstract

Resistance to 5-flucytosine (5-FC), used as an antifungal drug in combination therapy, compromises its therapeutic action. In this work, the response of the human pathogen *Candida glabrata* to 5-FC was evaluated at the membrane proteome level, using an iTRAQ-based approach. A total of 32 proteins were found to display significant expression changes in the membrane fraction of cells upon exposure to 5-FC, 50% of which under the control of CgPdr1, the major regulator of azole drug resistance. These proteins cluster into functional groups associated to cell wall assembly, lipid metabolism, amino acid/nucleotide metabolism, ribosome components and translation machinery, mitochondrial function, glucose metabolism, and multidrug resistance transport. Given the obtained indications, the function of the drug:H+ antiporters CgFlr1 (ORF *CAGL0H06017g*) and CgFlr2 (ORF *CAGL0H06039g*) was evaluated. The expression of both proteins, localized to the plasma membrane, was found to confer flucytosine resistance. CgFlr2 further confers azole drug resistance. The deletion of *CgFLR1* or *CgFLR2* was seen to increase the intracellular accumulation of 5-FC, or 5-FC and clotrimazole, suggesting that these transporters play direct roles in drug extrusion. The expression of *CgFLR1* and *CgFLR2* was found to be controlled by the transcription factors CgPdr1 and CgYap1, major regulator of oxidative stress resistance.

## Introduction

Systemic fungal infections are a problem of increasing clinical significance, especially since the prophylactic and therapeutic use of antifungal drugs has led to an increased number of infections with drug resistant fungal pathogens (Fidel et al., 1999; Mishra et al., 2007).

The antifungal drug 5-flucytosine (5-FC) is a fluorinated pyrimidine which enters fungal cells through permeases (Ghannoum and Rice, 1999; Hope et al., 2004; Edlind and Katiyar, 2010) and is then converted, by cytosine deaminase, to its metabolically active form 5-fluorouracil (5-FU) (Ghannoum and Rice, 1999; Espinel-Ingroff, 2008; Edlind and Katiyar, 2010). This antifungal drug acts by inhibiting transcription, DNA replication and protein synthesis (Ghannoum and Rice, 1999; Espinel-Ingroff, 2008). The specificity of this antimycotic relies on the absence of cytosine deaminase in mammalian cells (Hope et al., 2004; Edlind and Katiyar, 2010). However, 5-FU is considered toxic, mostly due to the conversion of flucytosine to fluorouracil by gut bacteria (Vermes et al., 2000). Despite these side-effects, 5-FC is still used clinically, mostly in combination therapy (Espinel-Ingroff, 2008).

Resistance to 5-FC in clinically relevant *Candida* species develops rapidly under treatment (Sanglard and Odds, 2002). Resistance is often related to decreased drug uptake by the Fcy2 cytosine permease or decreased conversion of 5-FC to 5-FU or 5-FUMP by the Fcy1 and Fur1 enzymes (Kontoyiannis and Lewis, 2002; Papon et al., 2007; Espinel-Ingroff, 2008). Some epidemiological studies suggest, however, that resistance mechanisms, independent of the Fcy2-Fcy1-Fur1 pathway, may play an important role in this phenomenon (Zhang et al., 2002). For example, reduction of 5-FC intracellular accumulation, mediated by the Drug:H+ Antiporter (DHA) CgAqr1 (Costa et al., 2013a), or by the acquaglyceroporins CgFps1 and CgFps2 (Costa et al., 2015) were recently registered.

Given these observations, in this study the *Candida glabrata* response to 5-FC was analyzed at the membrane proteome level, using an iTRAQ-based approach. Among the obtained results, the concentration of the DHA transporter CgFlr1 was seen to increase in 5-FC challenged cells. The role of CgFlr1 (ORF *CAGL0H06017g*), and of its very close homolog CgFlr2 (ORF *CAGL0H06039g*) (Gbelska et al., 2006; Costa et al., 2014a), in the resistance to 5-FC was then analyzed.

The deletion of *CgFLR1* had been found to lead to increased susceptibility to benomyl, diamide, and menadione, but not to fluconazole (Chen et al., 2007). As for CgFlr2, it remained uncharacterized until this study (Costa et al., 2014a). Both proteins, however, are close homologs of the *Saccharomyces cerevisiae* Flr1 DHA transporter. Flr1 confers resistance to many unrelated chemical compounds as reviewed in Sá-Correia et al. (2009), its expression being highly responsive to chemical stress exposure (Brôco et al., 1999; Teixeira et al., 2008, 2010; Sá-Correia et al., 2009; Dos Santos et al., 2014). The *FLR1* homolog in *Candida albicans, MDR1*, has been one of the few DHA transporters linked so far to azole drug resistance (White, 1997; Alarco and Raymond, 1999; Costa et al., 2013a,b, 2014a,b), being an important determinant of clinical acquisition of resistance against these antifungals (White, 1997; Alarco and Raymond, 1999; Costa et al., 2014a). These previous findings were used to guide the functional analysis of CgFlr1 and CgFlr2 in this study, in which the sub-cellular localization, role in antifungal drug resistance, and expression analysis was carried out.

## Materials and methods

### Strains and growth media

*C. glabrata* parental strain KUE100 (Ueno et al., 2007) and derived single deletion mutants KUE100_Δ*cgflr1* or KUE100_Δ*cgflr2*, constructed in this study, as well as the *C. glabrata* strains 66032u and 66032u_Δ*cgpdr1* (Vermitsky and Edlind, 2004), provided by Thomas Edlind, from Drexel University, College of Medicine, Philadelphia, PA, were batch-cultured at 30°C, with orbital agitation (250 rpm) in basal medium (BM), with the following composition (per liter): 1.7 g yeast nitrogen base without amino acids or NH4^+^ (Difco), 20 g glucose (Merck) and 2.65 g (NH4)_2_SO4 (Merck). *C. glabrata* strains L5U1 (*cgura3*Δ*0, cgleu2*Δ*0*), 84u (*cgura3*Δ*0*), and 84u_Δ*cgyap1*, kindly provided by John Bennett, (Chen et al., 2007) from the National Institute of Allergy and Infectious Diseases, NIH, Bethesda, USA, were grown in BM medium supplemented with 20 mg/L uracil and 60mg/L leucine. *S. cerevisiae* parental strain BY4741 (*MATa, ura3*Δ*0, leu2*Δ*0, his3*Δ*1, met15*Δ*0*) and the derived single deletion mutant BY4741_Δ*flr1* were obtained from Euroscarf (http://web.uni-frankfurt.de/fb15/mikro/euroscarf/). Cells were batch-cultured at 30°C, with orbital agitation (250 rpm) in supplemented with 20 mg/L methionine, 20 mg/L histidine, 60 mg/L leucine, 20 mg/L uracil (all from Sigma). Solid media contained, besides the above-indicated ingredients, 20 g/L agar (Iberagar). The plasmid pGREG576 was obtained from the Drag&Drop collection (Jansen et al., 2005).

### Membrane proteome-wide analysis of *C. glabrata* response to 5-flucytosine

Exponentially growing wild-type 66032 *C. glabrata* strain and the derived 66032_Δ*cgpdr1* deletion mutant were transferred to fresh BM medium in the absence of stress (control conditions) or in the presence of 4 μg/mL 5-flucytosine (Sigma). Upon 1 h of cultivation, cells were harvested by centrifugation and the membrane protein fraction was obtained as described before (Pais et al., 2016). Expression proteomics analysis of the obtained membrane-enriched fraction was carried out using an iTRAQ-MS procedure, carried out as a paid service at the Keck Foundation Biotechnology Resource Laboratory, Yale University, USA (http://medicine.yale.edu/keck/proteomics/index.aspx), using the method followed in Pais et al. (2016). Briefly, samples were sonicated and proteins reduced by adding 50 mM TCEP (tris(2-carboxyethyl)phosphine), followed by 200 mM MMTS (methyl methane thiosulfonate). Protein digestion was achieved by adding 10 μL of a solution of 1 mg/mL Lys-C, followed by incubation at 37°C for 3 h, and 10 μL of 1 mg/mL trypsin, followed by overnight incubation at 37°C. Macro-spin desalt of the digests with C18 spin columns for cleanup and quantitation was carried out, followed by dissolution in 65 μL of 500 mM TEAB. iTRAQ labeling was carried out based on the AAA quant protocol. iTRAQ experiments were carried out through the SCX cartridge and experiments run on 5600.

The search parameters and acceptance criteria used were the following: Peaklist generating software: ProteinPilot 4.5 and Mascot; Search engine: Paragon Search Engine (ProteinPilot 4.2); Sequence Database/spectral library: *C*. *glabrata* [5478] from SwissProt (May 2013); The database used was downloaded from UniProt, with a total of 5197 protein entries. Mass spectrometric analysis is done on an AB SCIEX TripleTOF® 5600 mass spectrometer with AB SCIEX ProteinPilot™ software used for protein identification and quantitation. ProteinPilot utilizes a Paragon™ algorithm with hybrid sequence tag and feature probability database searches. Hence, specific details such as mass tolerances, specific modifications etc., are not utilized. All iTRAQ results are uploaded into the Yale Protein Expression database (YPED) for investigator viewing. Protein identification was considered reliable for a Protein Score >2, corresponding to a confidence level of 99%. A reserve decoy database search, followed by filtering of all peptides above 1% False Discovery Rate was carried out before protein grouping.

Proteomics data analysis started from 3 iTRAQ sets. The samples present in each of the sets were randomized to prevent bias, and in different sets distinct labels were used to tag the samples, ensuring that protein identification in the MS step is not biased by the tags. For each sample in a given set, protein quantification was only considered for *p* < 0.05. Protein expression changes above 1.5-fold or below 0.66-fold were considered relevant. Protein classification into functional groups was achieved based on their predicted function, according to the Candida Genome Database (www.candidagenome.org), or based on the function of their closest *S. cerevisiae* homolog, according to the Saccharomyces Genome Database (www.yeastgenome.org).

### Disruption of the *CgFLR1* and *CgFLR2* genes

The deletion of the *C. glabrata FLR1* and *FLR2* genes (ORFs *CAGL0H06017g* and *CAGL0H06039g*, respectively) was carried out in the parental strain KUE100, using the method described by Ueno et al. (2011). The target genes *CgFLR1* and *CgFLR2* were replaced by a DNA cassette including the *CgHIS3* gene, through homologous recombination. The replacement cassette was prepared by PCR using the primers 5′ AGAGAAAAATAAAACCAATTCTAAAACCAAATCCATATTACAACCCAATTGCAAAGGGCCGCTGATCACG-3′ and 5′-AATGTTAGTGTGAACTTGAATGTTAGATTTTCACGTGAATGAGAACTGAGAAATCACATCGTGAGGCTGG-3′, for the *CgFLR1* gene, and the primers 5′-AGAATCATATTCATAAAGGTAACAAAACTACACAACAAATTATTAACTATTTTACAGGCCGCTGATCACG-3′ and 5′-AAATAATTTGTTCGGGGTAAGCACAATTGGAGGCTCTATCTTTTTTCTCTTCTTCACATCGTGAGGCTGG-3′, for the *CgFLR2* gene. The pHIS906 plasmid including *CgHIS3* was used as a template and transformation was performed as described previously (Ueno et al., 2007). Recombination locus and gene deletion were verified by PCR using the following pairs of primers: 5′-GAGGTGCTTAATATCGTCAC -3′ and 5′-CAACAACGTGTCCTACATG-3′; and 5′-GTGCATTTCAGGACACACT-3′ and 5′-GTATTTGTTCTTGTCCTGGTGTG -3′, respectively.

### Cloning of the *C. glabrata CgFLR1* and *CgFLR2* genes (ORFs *CAGL0H06017g* and *CAGL0H06039g*, respectively)

The pGREG576 plasmid from the Drag & Drop collection was used to clone and express the *C. glabrata* ORFs CAGL0H06017g and CAGL0H06039g in *S. cerevisiae*, as described before for other heterologous genes (Costa et al., 2013a,b, 2014b). *CgFLR1* or *CgFLR2* DNA was generated by PCR, using genomic DNA extracted from the sequenced CBS138 *C. glabrata* strain, and the following specific primers: 5′–GAATTCGATATCAAGCTTATCGATACCGTCGACAGCAAAGATGAATTATCTTC -3′ and 5′-GCGTGACATAACTAATTACATGACTCGAGGTCGACTCACCTGTTGTATTTAGACATGG -3′; or 5′-GAATTCGATATCAAGCTTATCGATACCGTCGACAATGTATATCGGTGCATTTCAGGAC -3′ and 5′-GCGTGACATAACTAATTACATGACTCGAGGTCGACTCATGAATCTGGACTAAATCTTG -3′, respectively. These plasmids include a *S. cerevisiae* CEN/ARS element which had been shown before to work in *C. glabrata* (Willins et al., 2002). The GAL1 promoter present in the pGREG576_*CgFLR1* and pGREG576_*CgFLR2* plasmids was then replaced by the copper-induced MTI *C. glabrata* promoter, giving rise to the pGREG576_MTI_*CgFLR1* and pGREG576_MTI_*CgFLR2* plasmids. The MTI promoter DNA was generated by PCR, using genomic DNA extracted from the sequenced CBS138 *C. glabrata* strain, and the following specific primers: 5′-TTAACCCTCACTAAAGGGAACAAAAGCTGGAGCTC*TGTACGACACGCATCATGTGGCAATC* -3′ and 5′-GAAAAGTTCTTCTCCTTTACTCATACTAGTGCGGC*TGTGTTTGTTTTTGTATGTGTTTGTTG* -3′. The recombinant plasmids pGREG576_*CgFLR1*, pGREG576_*CgFLR2*, pGREG576_MTI_*CgFLR1*, and pGREG576_MTI_*CgFLR2* were obtained through homologous recombination in *S. cerevisiae* and verified by DNA sequencing. As before (Costa et al., 2013a,b, 2014b), the transformation of L5U1 *C. glabrata* cells with the pGREG576 plasmids, as well as plasmid propagation was ensured by growth in selective uracil depleted medium.

### CgFlr1 and CgFlr2 subcellular localization assessment

The sub-cellular localization of the CgFlr1 and CgFlr2 proteins was determined based on the observation of BY4741 *S. cerevisiae* or L5U1 *C. glabrata* cells transformed with the pGREG576-*CgFLR1* and pGREG576-*CgFLR2* or pGREG576-MTI-*CgFLR1* and pGREG576-MTI-*CgFLR2* plasmids, respectively. These cells express the CgFlr1_GFP or CgFlr2_GFP fusion proteins, whose localization may be determined using fluorescence microscopy. *S. cerevisiae* cell suspensions were prepared by cultivation in BM-U medium, containing 0.5% glucose and 0.1% galactose, at 30°C, with orbital shaking (250 rpm), until a standard culture OD_600nm_ = 0.4 ± 0.04 was reached. At this point cells were transferred to the same medium containing 0.1% glucose and 1% galactose, to induce protein expression. *C. glabrata* cell suspensions were prepared in BM-U medium, until a standard culture OD_600nm_ = 0.4 ± 0.04 was reached, and transferred to the same medium supplemented with 50 μM CuSO_4_ (Sigma), to induce the expression of the fusion protein. After 5 h of incubation, the distribution of CgFlr1_GFP or CgFlr2_GFP fusion proteins in *S. cerevisiae* or in *C. glabrata* living cells was detected by fluorescence microscopy in a Zeiss Axioplan microscope (Carl Zeiss MicroImaging), using excitation and emission wavelength of 395 and 509 nm, respectively. Fluorescence images were captured using a cooled CCD camera (Cool SNAPFX, Roper Scientific Photometrics).

### Antifungal susceptibility assays

The susceptibility of the parental strain KUE100 toward toxic concentrations of the selected drugs was compared to that of the deletion mutants KUE100_Δ*cgflr1* and KUE100_Δ*cgflr2* by spot assays, using the steps described elsewhere (Costa et al., 2013b). The ability of *CgFLR1* and *CgFLR2* gene expression to increase wild-type resistance to the tested chemical stresses was also examined in the URA3- strain L5U1 *C. glabrata* strain, using the pGREG576_MTI_*CgFLR1* and pGREG576_MTI*_CgFLR2* centromeric plasmids. Additionally, the effect of *CgFLR1*, and *CgFLR2* expression in *S. cerevisiae* BY4741 wild-type strain and BY4741_Δ*flr1* single deletion mutant was also carried out as described elsewhere (Costa et al., 2013b). The tested drugs included the following compounds, used in the specified concentration ranges: the azole antifungal drugs ketoconazole (10–60 mg/L), fluconazole (75–250 mg/L), miconazole (0.10–0.50 mg/L), tioconazole (0.2–0.9 mg/L), itraconazole (0.1–20 mg/L), and clotrimazole (1–15 mg/L), the polyene antifungal drug amphotericin B (0.10–0.30 mg/L), the fluoropyrimidine 5-flucytosine (0.01–4 mg/L) and the pesticide mancozeb (0.5–2.5 mg/L) (all from Sigma).

### Drug accumulation assays

The internal accumulation of flucytosine or clotrimazole was determined by calculating the ratio between the radiolabeled compound measured within the yeast cells and in the external medium (Intracellular/Extracellular). The parental strain KUE100 and the mutant strains KUE100_Δ*cgflr1* and KUE100_Δ*cgflr2* were grown in BM medium until mid-exponential phase and harvested by filtration. Cells were washed and resuspended in BM medium, to obtain dense cell suspensions (OD_600nm_ = 0.5 ± 0.1, equivalent to ~ 1.57 mg (dry weight) mL^−1^). Readily, 0.1 μM of ^3^H- flucytosine or ^3^H- clotrimazole (American Radiolabelled Chemicals; 1 mCi/ml) and sub-inhibitory concentrations of the corresponding cold drug were added to the cell suspensions. Incubation proceeded for an additional period of 30 min. The intracellular accumulation of labeled antifungal was followed by filtering 200 μl of cell suspension, at adequate time intervals, through pre-wetted glass microfiber filters (Whatman GF/C). The filters were washed with ice-cold TM buffer and the radioactivity measured in a Beckman LS 5000TD scintillation counter. Extracellular ^3^H-drug was estimated, by radioactivity assessment of 50 μl of the supernatant. Non-specific ^3^H-drug adsorption to the filters and to the cells (<5% of the total radioactivity) was assessed and taken into consideration. To calculate the intracellular concentration of labeled antifungal drug, the internal cell volume (Vi) of the exponential cells, grown in the absence of drug and used for accumulation assays, was considered constant and equal to 2.5 μl (mg dry weight)^−1^ (Rosa and Sá-Correia, 1996). Statistical analysis of the results was performed using analysis of variance, and differences were considered significant for *P* < 0.05.

### *CgFLR1* and *CgFLR2* expression measurements

The levels of *CgFLR1* and *CgFLR2* transcripts were assessed by real-time PCR, using the approach described before (Costa et al., 2013b). Synthesis of cDNA for real time RT-PCR experiments, from total RNA samples, was performed using the Multiscribe™ reverse transcriptase kit (Applied Biosystems) and the 7500 RT-PCR Thermal Cycler Block (Applied Biosystems), following the manufacturer's instructions. The quantity of cDNA for the following reactions was kept around 10 ng. The subsequent RT-PCR step was carried out using SYBR® Green reagents. Primers for the amplification of the *CgFLR1, CgFLR2*, and *CgACT1* cDNA were designed using Primer Express Software (Applied Biosystems) and are—TCTTATTCACGATGCTACAAATTGG -3′ and 5′-GAATCACAAGGCCAGCAAAGTT -3′; 5′-GCAGCGGCATTCCCATTAT -3′ and 5′-CGGGATACTTTTTTGTGCTCAAT -3′; and 5′-AGAGCCGTCTTCCCTTCCAT -3′ and 5′-TTGACCCATACCGACCATGA -3′, respectively. The RT-PCR reaction was carried out using a thermal cycler block (7500 Real-Time PCR System—Applied Biosystems). Default parameters established by the manufacturer were used and fluorescence detected by the instrument and registered in an amplification plot (7500 System SDS Software—Applied Biosystems). The *CgACT1* mRNA level was used as an internal control. The relative values obtained for the wild-type strain in control conditions were set as 1 and the remaining values are presented relative to that control. To avoid false positive signals, the absence of non-specific amplification with the chosen primers was confirmed by the generation of a dissociation curve for each pair of primers. Statistical analysis of the results was performed using analysis of variance, and differences were considered significant for *P* < 0.05.

## Results

### Membrane proteome-wide changes occurring in response to flucytosine in *C. glabrata*

Given the importance of membrane proteins as a first line of defense against external stress agents, the membrane proteome of *C. glabrata* cells exposed to flucytosine-induced stress was compared to that of unstressed cells. Details on the protein quantification can be assessed at the Mass spectrometry Interactive Virtual Environment (MassIVE) repository (https://massive.ucsd.edu/ProteoSAFe/static/massive.jsp; MassIVE ID: MSV000079209). Annotated spectra for single peptide identification for each protein in the membrane-associated proteome is provided in MS-viewer (http://prospector2.ucsf.edu/prospector/cgi-bin/msform.cgi?form=msviewer; Search keys: qrvm65goix; xffxn22szm; tm0y9x0hqd) (Pais et al., 2016).

**Figure 1:**
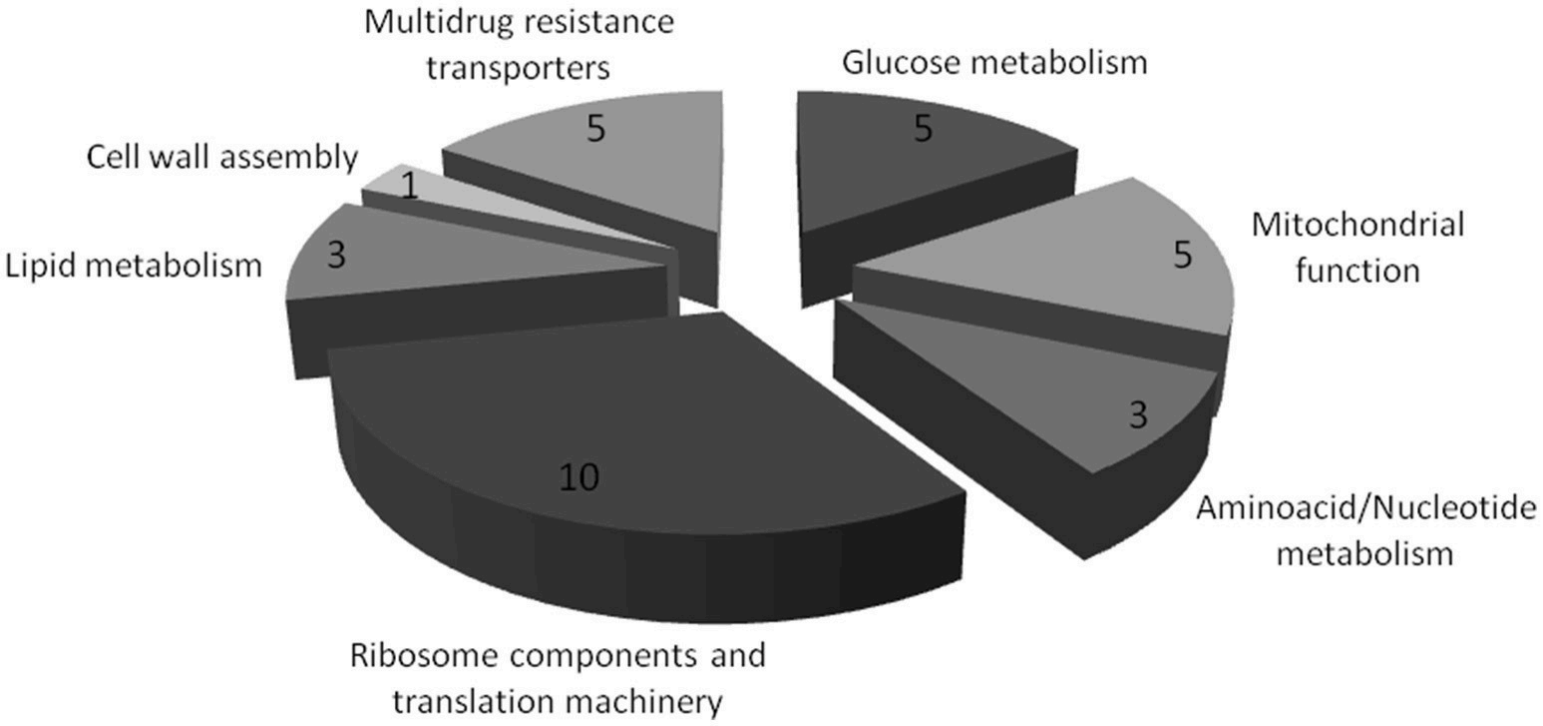
**Major functional groups found to have significant expression changes in the *C. glabrata* membrane-enriched proteome upon exposure to 5-flucytosine**. Proteins with significant expression changes include glucose metabolism (5 proteins), mitochondrial function (5 proteins), aminoacid/nucleotide metabolism (3 proteins), ribosome components and translation machinery (10 proteins), lipid metabolism (3 proteins), cell wall assembly (1 protein), and multidrug resistance transporters (5 proteins).

Among the identified membrane-associated proteins, 32 were found to exhibit more than 1.5-fold increased or decreased concentrations in *C. glabrata* cells exposed to inhibitory concentrations of flucytosine, when compared to the same cells growing in the absence of stress. Within these proteins, 21 were found to be down-regulated and 11 up-regulated in flucytosine challenged cells. Categorization of these proteins, based on their function predicted by homology to their *S. cerevisiae* homologs, enabled their clustering into seven groups: Glucose metabolism; Mitochondrial function; Amino acid/Nucleotide metabolism; Ribosome components and translation machinery; Lipid metabolism; Cell wall assembly; Multidrug Resistance transporters (Figure 1; Table 1).

**Table 1:** **Set of 32 proteins found to have significant expression changes in *C. glabrata* wild-type cells in the presence of flucytosine and correspondent fold changes in Δ*cgpdr1* mutant cells upon exposure to the drug**.

***C. glabrata*** **protein (ORF) name**	***S. cerevisiae*** **homolog**	**Description of the function of the *C. glabrata* protein or of its *S. cerevisiae* homolog**	**Wild-type fold change (upon 5-flucytosine stress)**	**Δ*****cgpdr1*** **fold change (upon 5-flucytosine stress)**
**GLUCOSE METABOLISM**				
*CgPDC1 (CAGL0M07920g)*	*PDC1*	Pyruvate decarboxylase, involved in pyruvate metabolism	0.54	0.39
*CAGL0L01485g*	*GSF2*	Putative protein of the ER membrane involved in hexose transporter secretion	0.60	0.43
*CgPGK1 (CAGL0L07722g)*	*PGK1*	Putative 3-phosphoglycerate kinase	0.20	0.63
*CgFBA1 (CAGL0L02497g)*	*FBA1*	Fructose-bisphosphate aldolase	0.58	0.47
*CAGL0G06138g*	*YCK1*	*S. cerevisiae* ortholog encodes a palmitoylated plasma membrane-bound casein kinase I isoform; functions in morphogenesis, endocytic trafficking, and glucose sensing	0.44	0.90^*^
**MITOCHONDRIAL FUNCTION**				
*CgRIP1 (CAGL0I03190g)*	*RIP1*	Putative ubiquinol-cytochrome C reductase iron-sulfur protein	1.68	0.28
*CAGL0F04213g*	*AAC2*	*S. cerevisiae* ortholog encodes a major ADP/ATP carrier of the mitochondrial inner membrane	0.64	1.00^*^
*CAGL0C02695g*	*MDM10*	Ortholog(s) have role in establishment of mitochondrion localization, mitochondrial outer membrane translocase complex assembly, phospholipid transport, protein import into mitochondrial outer membrane	0.51	0.91^*^
*CAGL0L06490g*	*PHB2*	Ortholog(s) have role in mitochondrion inheritance, negative regulation of proteolysis, protein folding and replicative cell aging	0.63	0.85^*^
*CAGL0M09713g*	*YIM1*	Putative protein involved in DNA damage response	0.24	0.48
**AMINO ACID / NUCLEOTIDE METABOLISM**				
*CgILV5 (CAGL0B03047g)*	*ILV5*	Ketol-acid reducto-isomerase	0.50	0.70^*^
*CgURA3 (CAGL0I03080g)*	*URA3*	Orotidine 5'-phosphate decarboxylase, catalyzes a step in pyrimidine biosynthesis; converts 5-FOA into 5-fluorouracil, a toxic compound	0.44	2.92
*CAGL0M12881g*	*URA1*	Ortholog(s) have dihydroorotate oxidase (fumarate) activity, role in 'de novo' pyrimidine nucleobase biosynthetic process	0.26	2.62
**RIBOSOME COMPONENTS AND TRANSLATION MACHINERY**				
*CAGL0L03846g*	*DBP2*	Ortholog(s) have RNA-dependent ATPase activity and role in mRNA catabolic process, nonsense-mediated decay, rRNA processing	3.94	2.15
*CAGL0E03938g*	*RPL4B*	*S. cerevisiae* ortholog encodes a ribosomal 60S subunit protein L13B	1.56	1.30^*^
*CAGL0K07414g*	*RPL20B*	*S. cerevisiae* ortholog encodes a ribosomal 60S subunit protein L20A	1.70	0.97^*^
*CAGL0J03234g*	*RPS24B*	Ortholog(s) have role in maturation of SSU-rRNA from tricistronic rRNA transcript	1.52	1.05^*^
*CAGL0K01859g*	*NOP1*	Ortholog(s) have mRNA binding, rRNA methyltransferase activity and role in box C/D snoRNA 3'-end processing, rRNA methylation	1.64	0.86^*^
*CAGL0I00792g*	*RPS16A*	Ortholog(s) have role in maturation of SSU-rRNA from tricistronic rRNA transcript (SSU-rRNA, 5.8S rRNA, LSU-rRNA) and 90S preribosome	1.97	1.27^*^
*CAGL0G01078g*	*RPL26A*	Ortholog(s) have RNA binding, structural constituent of ribosome activity, role in cytoplasmic translation and cytosolic large ribosomal subunit	1.78	1.37^*^
*CAGL0E02013g*	*RPL18A*	*S. cerevisiae* ortholog encodes a ribosomal 60S subunit protein L18A	0.59	0.48
*CAGL0L06886g*	*RPL13B*	*S. cerevisiae* ortholog encodes a ribosomal 60S subunit protein L13B	0.62	0.96^*^
*CAGL0A03278g*	*RPL19A*	*S. cerevisiae* ortholog encodes a ribosomal 60S subunit protein L19A	0.33	0.55
**LIPID AND CELL WALL METABOLISM**				
*CAGL0L03828g*	*CYB5*	Ortholog(s) have electron carrier activity, role in ergosterol biosynthetic process	2.14	0.98^*^
*CAGL0E03201g*	*CHO2*	Ortholog(s) have phosphatidylethanolamine N-methyltransferase activity, role in phosphatidylcholine biosynthetic process	1.56	1.56
*CAGL0M08206g*	*YJL171c*	*S. cerevisiae* ortholog encodes a GPI-anchored cell wall protein of unknown function; induced in response to cell wall damage	0.59	0.5
*CgHFD1 (CAGL0K03509g)*	*HFD1*	Putative mitochondrial fatty aldehyde dehydrogenase	0.29	0.24
**MULTIDRUG RESISTANCE TRANSPORTERS**				
*CgFLR1 (CAGL0H06017g)*	*FLR1*	Multidrug transporter of the major facilitator superfamily;	2.08	1.89^**^
*CgSNQ2 (CAGL0I04862g)*	*SNQ2*	Predicted plasma membrane ATP-binding cassette (ABC) transporter, putative transporter involved in multidrug resistance	0.61	0.75^*^
*CgCDR1 (CAGL0M01760g)*	*PDR5*	Multidrug transporter of ATP-binding cassette (ABC) superfamily, involved in resistance to azoles	0.30	0.1
*CgYOR1 (CAGL0G00242g)*	*YOR1*	Putative ABC transporter involved in multidrug efflux	0.51	0.44
*CgQDR2 (CAGL0G08624g)*	*QDR2*	Drug:H+ antiporter of the Major Facilitator Superfamily, confers imidazole drug resistance	0.57	0.31

*The name of the proteins whose expression change was found to vary more than 1.5-fold in the Δcgpdr1 mutant when compared to the wild-type is underlined. Protein description and clustering was obtained from the Candida Genome Database (www.candidagenome.org) or, when completely uncharacterized, based on the role of their predicted S. cerevisiae homologs, obtained from the Yeast Genome Database (www.yeastgenome.org)*.

* *Fold change value outside of the chosen cut-off intervals (0.67 < fold change < 1.5)*;

** *Fold change quantification considered as not statistically significant (p > 0.05)*.

The largest functional group identified in the 5-FC membrane proteome response, including a third of the proteins with altered content, is related to RNA metabolism. The expression of seven proteins involved in ribosome biogenesis and translation was found to increase in flucytosine stressed cells, whereas three proteins of the same category were found to be down-regulated in these conditions. It is difficult to say what the exact consequences of the altered expression of each of them individually may be. Additionally, the concentration of several proteins related to glucose metabolism and mitochondrial function related proteins was found to decrease in the presence of flucytosine. The expression of one amino acid biosynthetic protein, Ilv5, and two pyrimidine biosynthetic proteins, Ura1, and Ura3, was also found to decrease in cells exposed to this antifungal agent. Interestingly, Ura3 is responsible for the conversion of 5-FOA into 5-fluorouracil, a key step in the conversion of flucytosine into its toxic sub-products. The repressed expression of the Ura proteins may confer an advantage to flucytosine stressed cells as it may delay the conversion of this pro-drug into its toxic subproducts. Finally, a group of five multidrug transporters was found to exhibit altered levels of expression in flucytosine stressed cells. Four of them, previously implicated in azole drug resistance (Sanglard et al., 1999; Vermitsky et al., 2006; Torelli et al., 2008; Costa et al., 2013b), were actually found to be down-regulated, while the fifth, CgFlr1, was found to be more than 2-fold up-regulated upon *C. glabrata* exposure to flucytosine.

### Effect of *CgPDR1* deletion in the membrane proteome-wide changes occurring in response to flucytosine in *C. glabrata*

**Figure 2:**
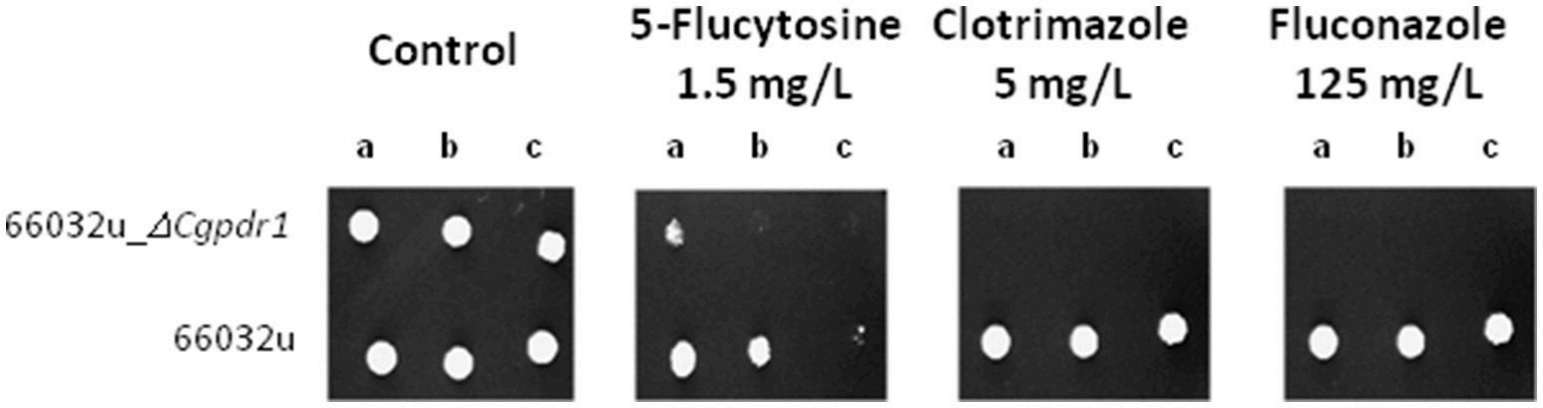
**CgPdr1 confers resistance to flucytosine in *C. glabrata* cells**. Comparison of the susceptibility to inhibitory concentrations of flucytosine, clotrimazole and fluconazole, at the indicated concentrations, of the *C. glabrata* 66032u and 66032u_Δ*cgpdr1* strains, in BM plates by spot assays. The inocula were prepared as described under “Materials and Methods.” Cell suspensions used to prepare the spots were 1:5 (b) and 1:25 (c) dilutions of the cells suspension used in (a). The displayed images are representative of at least three independent experiments.

To assess the possible involvement of the transcription factor Pdr1 in the resistance to 5-flucytosine, the growth of the *C. glabrata* strains 66032u and 66032u_Δ*cgpdr1* (Vermitsky and Edlind, 2004) was compared in solid medium containing inhibitory concentrations of flucytosine, clotrimazole and fluconazole (Figure 2). *CgPDR1* deletion fully abrogates growth in the presence of the tested azole drug concentrations, as expected based on numerous reports on the pivotal role of this transcription factor in azole drug resistance. Interestingly, the Δ*cgpdr1* deletion mutant was also found to display higher susceptibility to the antifungal drug 5-FC than the wild-type parental strain, suggesting that it plays a role in the resistance to this drug as well. Based on this result, the analysis of the membrane-enriched fraction of the *C. glabrata* proteome obtained from cells exposed to 5-flucytosine in the absence of the transcription factor CgPdr1 was assessed and compared to that of the *C. glabrata* wild-type cells exposed to 5-flucytosine. Considering an at least 1.5-fold difference in protein expression activation in wild-type vs. Δ*cgpdr1* cells exposed to 5-flucytosine, nine proteins were found to be repressed by CgPdr1; while eight proteins were found to be activated by CgPdr1 (Table 1). The PDRE loci BCCRYYRGD, TCCRYGGA (Tsai et al., 2010), TCCACGGA, and HYCCGTGGR (Paul et al., 2014), were searched for in the promoters of the referred genes using the PathoYeastract database (Monteiro et al., 2016). Interestingly, considering these 17 proteins, at least one CgPdr1-binding site is found in the promoter regions of nine genes, suggesting the action of CgPdr1 in their expression may be direct. For the remaining 15 proteins, no statistically significant change could be detected in the current experiment. The proteins whose expression was found to be higher in the wild-type strain than in the Δ*cgpdr1* deletion mutant include the multidrug transporters CgCdr1 and CgQdr2, but also two proteins related to mitochondrial function, CgCyb5 (ORF *CAGL0L03828g*) and CgRip1 (ORF *CAGL0I03190g*), and four proteins involved in RNA metabolism, CgDbp2 (ORF *CAGL0L03846g*), CgNop1 (ORF *CAGL0K01859g*), CgRpl20B (ORF *CAGL0K07414g*), and CgRps16A (ORF *CAGL0I00792g*).

### CgFlr1 and CgFlr2 expression confers resistance to other chemical stress inducers

**Figure 3:**
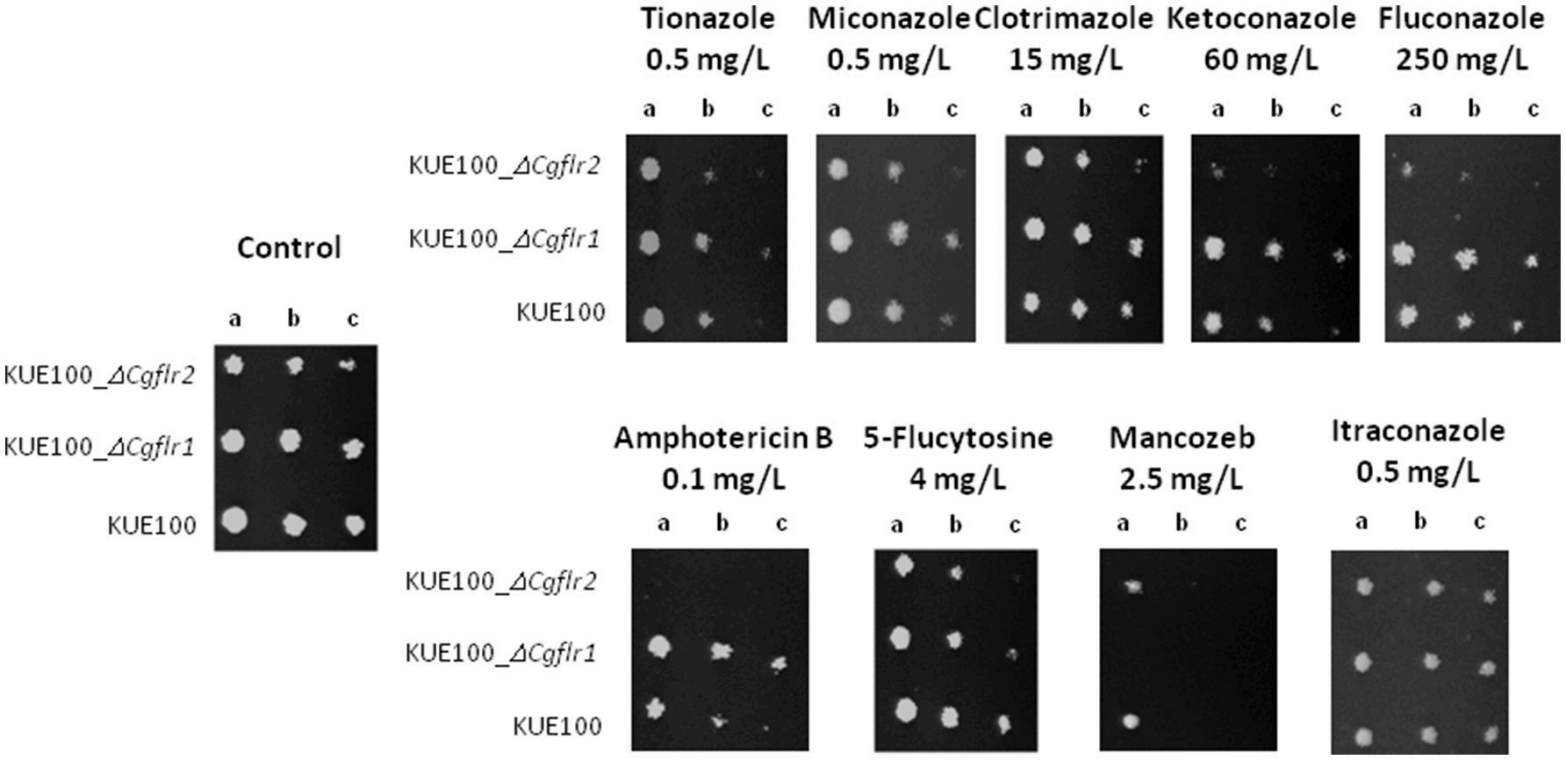
**CgFlr1 and CgFlr2 confer resistance to flucytosine in *C. glabrata* cells**. Comparison of the susceptibility to inhibitory concentrations of several chemical stress inducers, at the indicated concentrations, of the *C. glabrata* KUE100, KUE100_Δ*cgflr1* and KUE100_Δ*cgflr2* strains, in BM plates by spot assays. The inocula were prepared as described under “Materials and Methods.” Cell suspensions used to prepare the spots were 1:5 (b) and 1:25 (c) dilutions of the cells suspension used in (a). The displayed images are representative of at least three independent experiments.

The involvement of CgFlr1 and CgFlr2 in antifungal drug resistance was evaluated, through susceptibility assays, considering a total of nine antifungal drugs of four different families and one agricultural fungicide mancozeb. The results obtained by screening the susceptibility phenotypes of Δ*cgflr1* and Δ*cgflr2* mutants, when compared to the wild-type strain, reveal that *CgFLR1* confers resistance to mancozeb, whereas *CgFLR2* confers resistance to azoles and amphotericin B (Figure 3).

**Figure 4:**
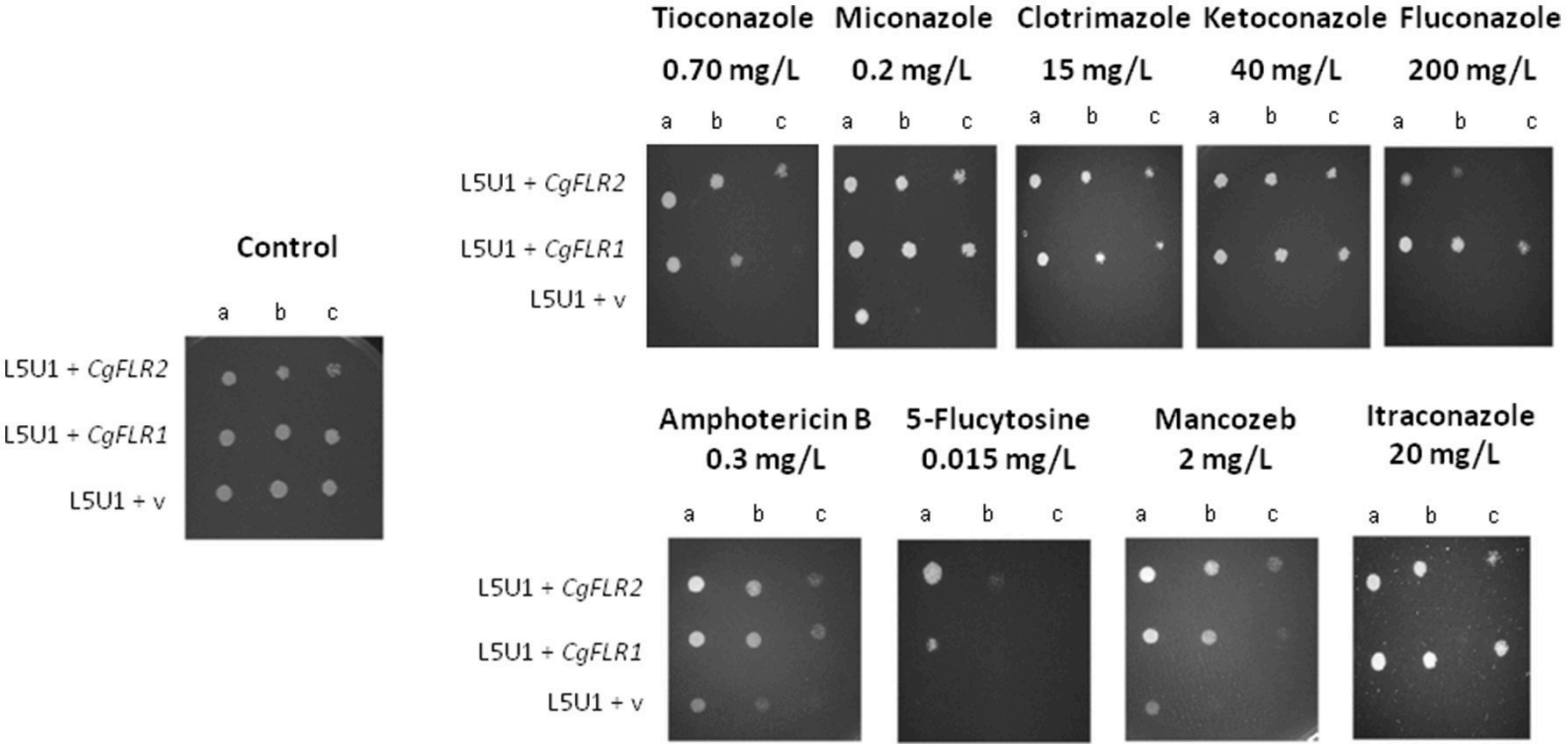
**CgFlr1 and CgFlr2 expression increases flucytosine resistance in *C. glabrata* cells**. Comparison of the susceptibility to inhibitory concentrations of several chemical stress inducers, at the indicated concentrations, of the *C. glabrata* L5U1 strain, harboring the pGREG576 cloning vector (v) or the pGREG576_MTI_*CgFLR1* or pGREG576_MTI_*CgFLR2* plasmids, in BM-U plates (50 μM CuSO_4_ supplemented) by spot assays. The inocula were prepared as described under “Materials and Methods.” Cell suspensions used to prepare the spots were 1:5 (b) and 1:25 (c) dilutions of the cells suspension used in (a). The displayed images are representative of at least three independent experiments.

Both *CgFLR1* and *CgFLR2* were found to confer resistance to flucytosine, although the effect of *CgFLR2* is stronger (Figure 3). The expression of *CgFLR1-GFP* or *CgFLR2-GFP* in the L5U1 *C. glabrata* wild-type strain, as verified by anti-GFP western analysis (Figure S1), was concordantly found to increase *C. glabrata* natural resistance toward the antifungal drugs to which the deletion of each gene was found to lead to a susceptibility phenotype (Figure 4).

**Figure 5:**
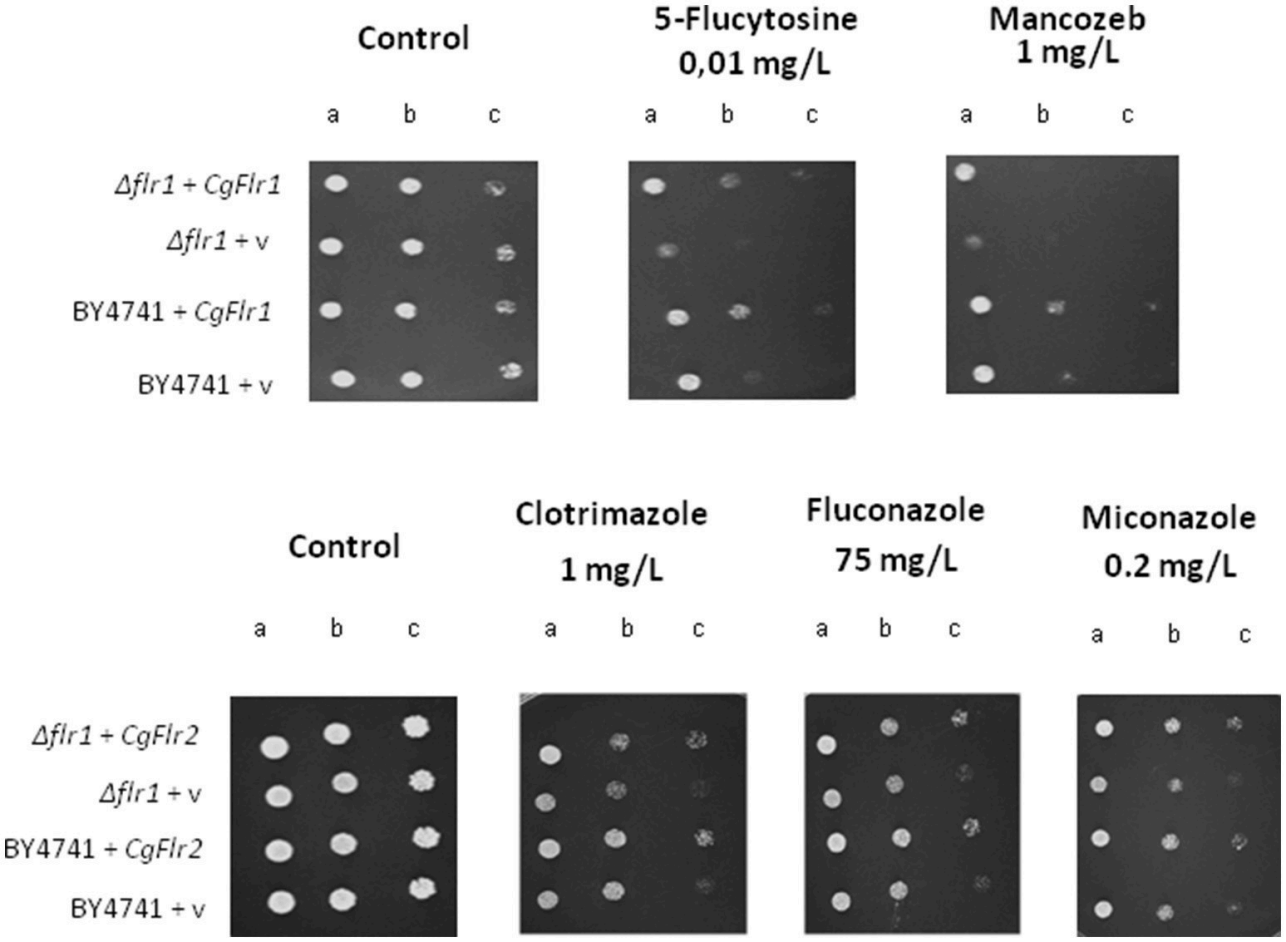
**CgFlr1 and CgFlr2 confer resistance to antifungal drugs when heterologously expressed in *S. cerevisiae* cells**. Comparison of the susceptibility to inhibitory concentrations of several chemical stress inducers, at the indicated concentrations, of the *S. cerevisiae* BY4741 and BY4741_Δ*flr1* strains, harboring the pGREG576 cloning vector (v) or the pGREG576_*CgFLR1* of pGREG576_*CgFLR2* plasmids, in BM-U plates by spot assays. The inocula were prepared as described under “Materials and Methods.” Cell suspensions used to prepare the spots were 1:5 (b) and 1:25 (c) dilutions of the cells suspension used in (a). The displayed images are representative of at least three independent experiments.

Using *S. cerevisiae* as a heterologous expression system, the effect of *CgFLR1* and *CgFLR2* expression on yeast drug resistance was further investigated. When expressed in *S. cerevisiae*, the *CgFLR1* and *CgFLR2* genes were found to rescue the susceptibility phenotype exhibited by the *S. cerevisiae* Δ*flr1* mutant against flucytosine and mancozeb, and azole drugs, respectively (Figure 5).

### CgFlr1 and CgFlr2 are localized to the plasma membrane

**Figure 6:**
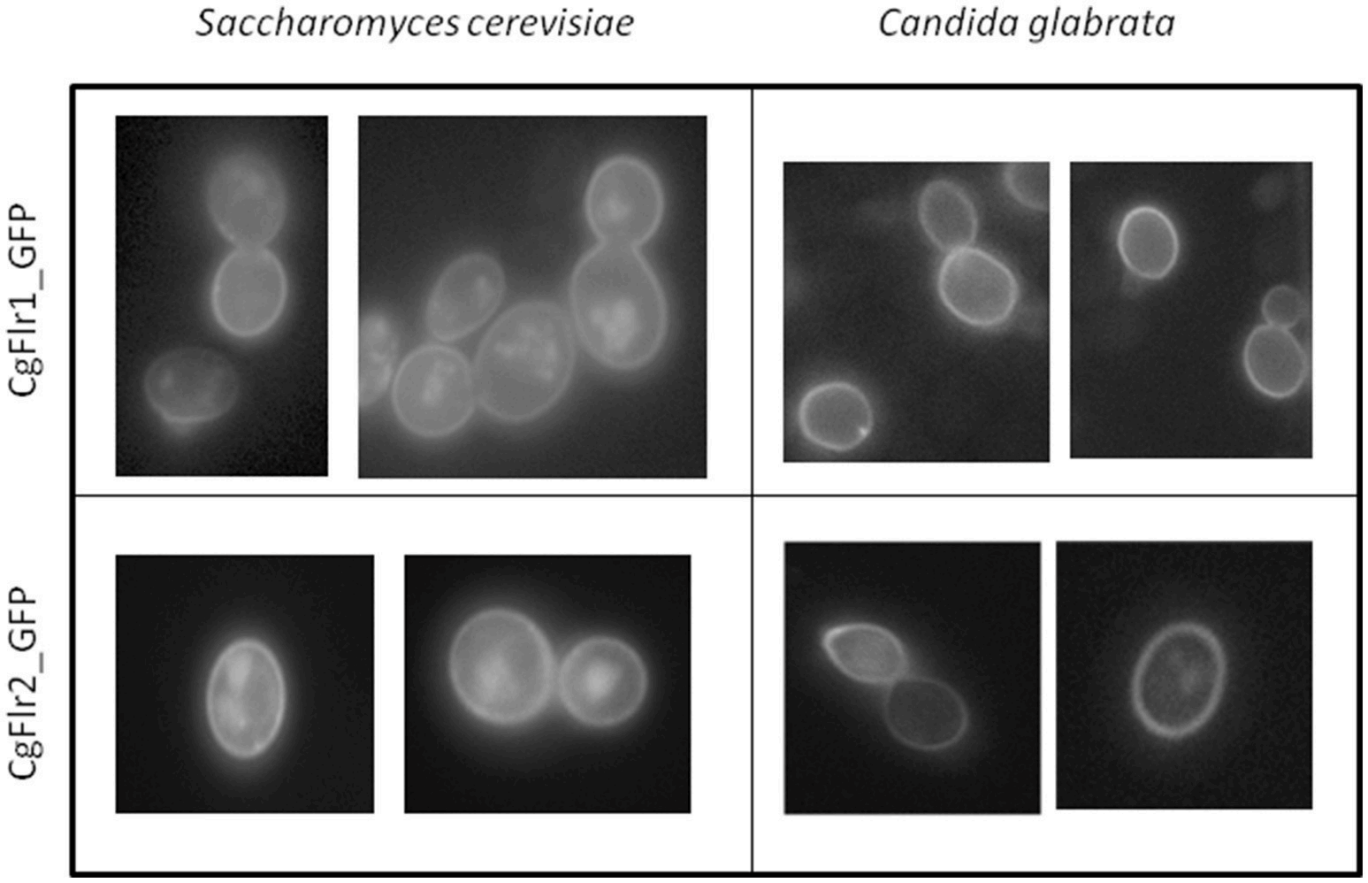
**CgFlr1 and CgFlr2 are plasma membrane proteins**. Fluorescence of exponential phase BY4741 *S. cerevisiae* and L5U1 *C. glabrata* cells, harboring the expression plasmids pGREG576_*CgFLR1* and pGREG576_*CgFLR2* or pGREG576_*MTI_CgFLR1*, and pGREG576_*MTI_CgFLR2*, after galactose or copper-induced recombinant protein production, respectively.

Upon expression induction, *C. glabrata* cells harboring the pGREG576_MTI*_CgFLR1* and pGREG576_MTI*_CgFLR2* plasmids were inspected by fluorescence microscopy. The CgFlr1_GFP and CgFlr2_GFP fusion proteins were found to be localized to the cell periphery (Figure 6). In a similar approach, CgFlr1_GFP and CgFlr2_GFP were found to be localized to the cell periphery when expressed heterologously in *S. cerevisiae* (Figure 6). These results strongly suggest plasma membrane localization for both CgFlr1 and CgFlr2.

### CgFlr1 and CgFlr2 reduce the intracellular accumulation of antifungal drugs in *C. glabrata*

**Figure 7:**
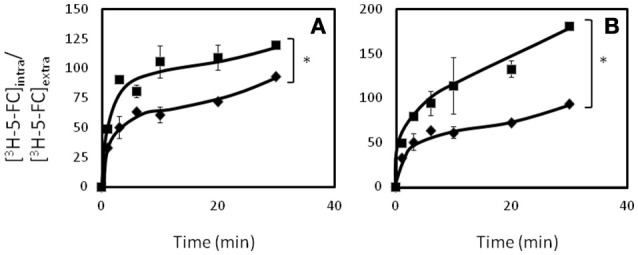
**CgFlr1 and CgFlr2 expression decreases the intracellular accumulation of ^3^H-flucytosine**. Time-course accumulation of radiolabeled ^3^H-flucytosine in KUE100 wild-type (♦) and KUE100_Δ*cgflr1* (■) **(A)** and KUE100 (♦) and KUE100_Δ*cgflr2* (■) **(B)** strains, during cultivation in BM liquid medium in the presence of sub-lethal concentrations of unlabeled flucytosine. Accumulation values are the average of at least three independent experiments. Errors bars represent the corresponding standard deviations. ^*^p < 0.05.

Consistent with the observed susceptibility phenotypes, Δ*cgflr1* and Δ*cgflr2* deletion mutants were found to accumulate 2-fold more radiolabeled flucytosine than the KUE100 parental strain (Figures 7A,B).

**Figure 8:**
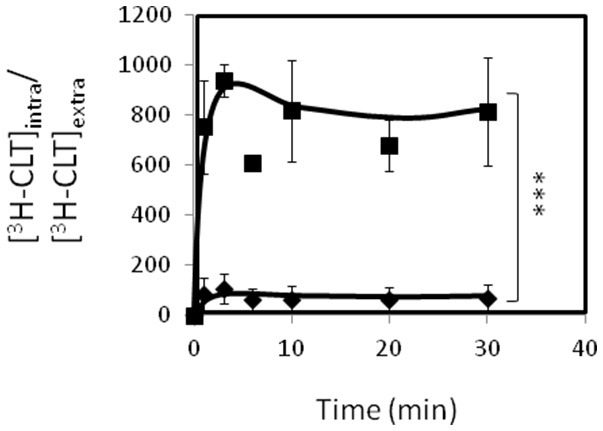
**CgFlr2 expression decreases the intracellular accumulation of ^3^H-clotrimazole**. Time-course accumulation of radiolabeled ^3^H-clotrimazole in KUE100 wild-type (♦) and KUE100_Δ*cgflr2* (■) strains, during cultivation in BM liquid medium in the presence of sub-lethal concentrations of unlabeled clotrimazole. Accumulation values are the average of at least three independent experiments. Errors bars represent the corresponding standard deviations. ^***^p < 0.001.

Additionally, Δ*cgflr2* mutant cells were found to accumulate around 8-fold more radiolabelled clotrimazole than the parental strain (Figure 8). These results strongly suggest that CgFlr1 and CgFlr2 activities increase *C. glabrata* resistance toward flucytosine, and in the case of CgFlr2 toward azoles, by reducing their accumulation in yeast cells.

### *CgFLR1* and *CgFLR2* transcript levels are up-regulated under antifungal stress, their basal expression being controlled by CgPdr1 and CgYap1 transcription factors

**Figure 9:**
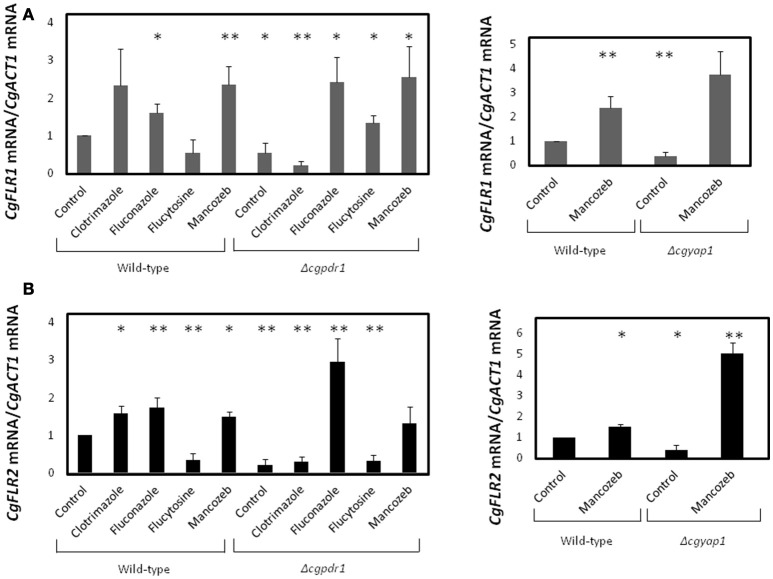
***CgFLR1*** **and *CgFLR2* transcriptional control**. Comparison of the variation of the *CgFLR1* **(A)** and *CgFLR2* **(B)** transcript levels in the 66032u *C. glabrata* wild-type strain and in the derived 66032u_Δ*cgpdr1* deletion mutant; and in the 84u *C. glabrata* wild-type strain and in the derived 84u_Δ*cgyap1* deletion mutant, before and after 1 h of exposure to 60 mg/L clotrimazole, 80 mg/L fluconazole, 8 mg/L flucytosine and 20 mg/L mancozeb. The presented transcript levels were obtained by quantitative RT-PCR and are relative *CgFLR1/CgACT1* or *CgFLR2/CgACT1* mRNA, relative to the values registered in the 66032 or 84u parental strains in control conditions. The indicated values are averages of at least three independent experiments. Error bars represent the corresponding standard deviations. ^*^p < 0.05; ^**^p < 0.01.

In order to evaluate whether or not the expression of *CgFLR1* and *CgFLR2* is affected upon drug exposure, quantitative RT-PCR was used to study the effect of flucytosine, clotrimazole, and mancozeb stress exposure in the transcript levels of these genes. No up-regulation of *CgFLR1* or *CgFLR2* genes could be detected in wild-type cells upon flucytosine exposure. Interestingly, however, *CgFLR1* was found to be up-regulated in mancozeb exposed cells, whereas both *CgFLR1* and *CgFLR2* were found to be up-regulated upon exposure to clotrimazole or fluconazole stress, which is consistent with the role of *CgFLR1* and *CgFLR2* in mancozeb and azole drug resistance, respectively (Figures 9A,B).

Additionally, given the fact that the transcription factor CgPdr1 is the master regulator of azole drug resistance (Tsai et al., 2006) and CgYap1 had been previously linked to the control of CgFlr1 expression (Chen et al., 2007), the effect of CgYap1 and CgPdr1 deletion in the control of the expression of *CgFLR1* and *CgFLR2* was further evaluated. *CgFLR1* and *CgFLR2* up-regulation, registered under clotrimazole exposure—but not under fluconazole stress —, was found to be abrogated in the absence of *CgPDR1* (Figure 9B), while CgPdr1 and CgYap1 were found to control the basal expression of both *CgFLR1* and *CgFLR2* (Figures 9A,B). As expected, based on the proteomics data, none of the genes was found to be controlled by CgPdr1 in flucytosine exposed cells. Unexpectedly, the up-regulation of *CgFLR1*, and *CgFLR2* registered under mancozeb stress was found not to be controlled by CgYap1. Indeed, in Δ*cgyap1* cells the mancozeb-induced up-regulation of these genes was found to be even stronger than that registered in the wild-type strain (Figures 9A,B).

## Discussion

5-Flucytosine has fallen into disuse due to the rapid acquisition of resistance by fungal pathogens and to its moderate toxicity in humans, limiting the administration of higher dosages. The identification of the mechanisms underlying these phenomena is, thus, crucial to maintain the use of 5-FC as a therapeutic option. In this work, the changes occurring at the membrane proteome level in *C. glabrata* cells exposed to 5-FC were analyzed, highlighting new mechanisms of resistance to this antifungal drug.

One of the most interesting aspects of this work concerns the identification of CgPdr1 transcription factor, a major factor of resistance to azoles (Tsai et al., 2006), as a determinant of 5-FC resistance. The deletion of CgPdr1 was consistently found to decrease the activation of about 50% of the membrane proteins found to be up-regulated in response to 5-FC. Among the proteins whose expression was seen to be affect by CgPdr1 deletion are the multidrug efflux transporter encoding genes *CgCDR1* and *CgQDR2*, but also *CgRIP1*, encoding a putative ubiquinol-cytochrome C reductase iron-sulfur protein, which were previously found to be controlled by this transcription factor (Tsai et al., 2006; Vermitsky et al., 2006; Ferrari et al., 2011; Costa et al., 2013b; Pais et al., 2016). New putative targets of CgPdr1 up-regulated in the context of 5-FC response include four proteins involved in RNA metabolism, CgDbp2, CgNop1, CgRpl20B, and CgRps16A. Interestingly, the traditional targets of CgPdr1, including the ABC drug efflux pumps CgCdr1, CgYor1, and CgSnq2 are repressed in response to 5-FC, which suggests that, although being active in the response to azoles and to 5-FC, the action of Pdr1 at the level of transcriptional control appears to be different. It will be interesting to test whether this differential outcome of CgPdr1 activity is linked to differences in terms of the conformation of this transcription factor, whose activation is known to occur by the direct binding to the drug molecule (Thakur et al., 2008). To gain a full view on the extension of the participation of CgPdr1 in 5-FC response, however, it would be necessary to conduct a transcriptomics study.

A large proportion of the 5-FC response was found to be related to RNA and protein metabolism. Interestingly, an increased expression of some ribosome and translation associated proteins was observed, which may be related to the specific mechanism of action of 5-FC. It is thus possible to assume that the RNA- and protein-metabolism-related genes identified herein as responding to 5-FC challenge may be involved in counteracting its primary toxic action. Previous analyses of the transcriptome-wide *S. cerevisiae* (Zhang et al., 2002) or *Candida albicans* (Liu et al., 2005) response to 5-FC, also highlighted the relevance of RNA metabolism in the response to this antifungal drug. Indeed, Liu et al. found an up-regulation of several genes involved in RNA metabolism and translation (Liu et al., 2005), while Zhang and co-workers showed a down regulation of a few ribosomal protein encoding genes in these conditions (Zhang et al., 2002). It is also in agreement with a previous chemogenomic analysis of the determinants of 5-FC resistance in the model yeast *S. cerevisiae*, in which about one fourth of the determinants of resistance to this drug were found to be related to RNA and protein metabolism (Costa et al., 2015). It appears that *C. glabrata* cells try to compensate, with the increased expression of translation associated proteins, the detrimental effect that 5-FC exerts in this process.

Another interesting feature of the proteomics response includes the down-regulation of two nucleotide biosynthesis related proteins, CgUra1 and CgUra3. CgUra1 catalyses the synthesis of ororate from dihydroorotate, which is, then converted to oritidine-5-phosphate. CgUra3 catalyses the next step of conversion of oritidine-5-phosphate to UMP, which is funneled into the production of UDP and UTP, used for RNA synthesis. This same pathway is used to process 5-FC into its toxic products, including 5F-UDP, which upon incorporation in RNA molecules inhibits protein synthesis. It appears, thus, that the cell responds to 5-FC induced stress by decreasing the expression of enzymes required for its conversion to toxic 5-FC products. It is interesting to point out, in this context, that the expression level of CgUra1 and CgUra3 in the Δ*cgpdr1* deletion mutant is much higher than in wild-type cells. The up-regulation of these proteins in the Δ*cgpdr1* background may underlie the increased susceptibility to 5-FC exhibited by this deletion mutant, when compared to the wild-type strain.

Among the results obtained from the membrane proteomics analysis, the role of CgFlr1 and of its homolog CgFlr2, in 5-FC response was further analyzed. Although the concentration of CgFlr2 was not found to be increased in the membrane proteome of *C. glabrata* cells, both CgFlr1, and CgFlr2 were found to confer resistance to 5-FC, apparently due to their role in controlling the levels of 5-FC accumulation within *C. glabrata* cells. The fact that CgFlr2 is not up-regulated in 5-FC-exposed cells but is required for *C. glabrata* resistance to this stress, although unexpected, is consistent with the frequent observation that the genes that are required for the resistance to a given stress are not necessarily up-regulated in response to that stress (Teixeira et al., 2011). Interestingly, CgFlr1 was further found to confer resistance to mancozeb, while CgFlr2 was also found to confer resistance to azoles and amphotericin B, placing this transporter at the intersection of multiple antifungal resistance mechanisms. These two transporters constitute, thus, two additional players in the antifungal drug resistance phenomenon. Our group had previously shown that the acquaglyceroporins CgFps1 and CgFps2 (Costa et al., 2015), as well as the DHA transporters CgAqr1 (Costa et al., 2013a) and CgTpo1_1 and CgTpo1_2 (Pais et al., 2016) are determinants of flucytosine resistance as well, suggesting that 5-FC extrusion is an important mechanism of resistance against this antifungal drug and showing this phenomenon to be the consequence of the additive contribution of several players.

The analysis of the expression of the *CgFLR1* and *CgFLR2* genes further highlighted their importance in the context of drug resistance in *C. glabrata*. Using the PathoYeastract database (http://pathoyeastract.org/cglabrata/index.php Monteiro et al., 2016), it is possible to verify that the *CgFLR1* was found to be up-regulated upon the over-expression of CgPdr1, in control conditions (Noble et al., 2013), or upon benomyl or selenite exposure, in the dependency of CgYap1 (Chen et al., 2007; Lelandais et al., 2008; Merhej et al., 2016). Additionally, *CgFLR1* expression was shown to be repressed by the transcription factor Stb5, a negative regulator of azole resistance in *C. glabrata* (Noble et al., 2013). Chromatine ImmunoPrecipitation (ChIP) assays further showed that Yap7 binds to the *CgFLR1* promoter in selenite exposed cells (Merhej et al., 2016). Information on the regulation of *CgFLR2* is much scarcer. RNA sequencing data have demonstrated that the predicted zinc cluster transcription factor encoded by ORF *CAGL0I07755g*, a homolog of the *S. cerevisiae* Hal9 and of the *C. albicans* Tac1 transcription factors, is a positive regulator of *CgFLR2* in control conditions (Wu et al., 2015). The new data on the regulation of the *CgFLR1* and *CgFLR2* genes coming from this study shows that both genes are controlled at the transcription level by CgPdr1 in the presence of the azole drug clotrimazole, but not in the presence of fluconazole. The fact that in previous genome-wide expression analysis of fluconazole response in *C. glabrata* the regulation of *CgFLR1* or *CgFLR2* by CgPdr1 had not been identified suggests that this effect may be specific to imidazole antifungals, such as clotrimazole, but not to triazole antifungals such as fluconazole. Despite the fact that a previous ChIP experiment probing CgPdr1 targets in ρ0 *C. glabrata* cells did not pinpoint *CgFLR1* or *CgFLR2* promoters as targets of CgPdr1 (Paul et al., 2014), a putative CgPdr1 binding site can be found in the promoter region of *CgFLR2*. The possibility, however, that CgPdr1 may regulate the expression of *CgFLR1* and *CgFLR2* in clotrimazole stressed cells through direct binding to their promoter regions remains to be established. Given the importance of CgPdr1 in the clinical acquisition of azole drug resistance, these results raise the hypothesis that CgFlr1 and CgFlr2 may be relevant in the clinical context. In a previous transcriptomics analysis of the impact of CgPdr1 GOF mutations in fluconazole resistant isolates, when compared to susceptible ones, no significant changes were detected in terms of the expression of *CgFLR1* or *CgFLR2* (Tsai et al., 2010). However, it would indeed be interesting to assess whether there is a possible correlation between the expression of these genes and the level of antifungal drug resistance in clinical isolates displaying differential clotrimazole or 5-FC susceptibility phenotypes. Additionally, the fact that the transcript levels of both *CgFLR1* and *CgFLR2* genes is controlled, at least at the basal level, by CgPdr1 and CgYap1, appears to correlate with the complex regulation of their *S. cerevisiae* homolog Flr1. The observation that *S. cerevisiae FLR1* gene is also strongly up-regulated by the transcription factor Yap1 in mancozeb stressed cells (Teixeira et al., 2008) urged us to check for a similar effect in *C. glabrata CgFLR1* and *CgFLR2* genes. However, although an increased expression of *CgFLR1* and *CgFLR2* was indeed registered in *C. glabrata* cells exposed to mancozeb, this proved to be irrespective of CgYap1 activity, suggesting that the control of the expression of these genes is not fully conserved in *C. glabrata*. The transcriptional control of ScFlr1 was found to be highly complex, relying on the combined efforts of at least four transcription factors, Yap1, Pdr1, Yrr1, and Rpn4 (Alarco et al., 1997; Brôco et al., 1999; Teixeira et al., 2008). The building of a mathematical model to describe the ScFlr1 regulatory network highlighted that this network is likely to require a fifth, still unidentified, transcription factor to explain the experimental observations, putting forward the hypothesis that its regulation may be even more complex than initially foreseen (Teixeira et al., 2010; Monteiro et al., 2011). It would be interesting to check whether the homologs in *C. glabrata* of these additional *S. cerevisiae* transcription factors may also be important in the regulation of these genes, whose control appears to be phylogenetically conserved among these yeast species.

In conclusion, the results described in this study highlight the importance of the DHA transporters from the MFS in antifungal resistance. This work highlights the importance of proteome-wide approaches in the identification of new antifungal resistance mechanisms. The newly identified processes stand out as promising targets for the development of new 5-flucytosine chemosensitizers, which would expectedly allow for the use of decreased therapeutic dosages of 5-flucytosine.

## Author contributions

PP and CP conducted most of the experiments and contributed to the writing of the manuscript. CC and MO contributed to development of some of the experimental work. HC and MT conceived the experiments. MT supervised the scientific and experimental design of the work and wrote the manuscript.

## Funding

This work was supported by FEDER and “Fundação para a Ciência e a Tecnologia” (FCT) (Contract PTDC/BBB-BIO/4004/2014 and Ph.D. and post-doctoral grants to PP (SFRH/BD/110956/2015) and CC (SFRH/BPD/100863/2014), respectively). Funding received by iBB-Institute for Bioengineering and Biosciences from FCT-Portuguese Foundation for Science and Technology (UID/BIO/04565/2013) and from Programa Operacional Regional de Lisboa 2020 (Project N. 007317) is acknowledged.

### Conflict of interest statement

The authors declare that the research was conducted in the absence of any commercial or financial relationships that could be construed as a potential conflict of interest.

## Supplementary material

Figure S1 **CgFlr1 and CgFlr2 are expressed in *C. glabrata* cells harboring the pGREG576_MT1_*CgFLR1* and the pGREG576_MT1_*CgFLR2* plasmids**. Comparison of the level of expression of CgFlr1-GFP or CgFlr2-GFP fusion proteins in L5U1 *C. glabrata* cells upon exposure to the indicated CuSO_4_ concentrations, based on anti-GFP immuno-detection. Cells harboring the pGREG576 cloning vector (control), exposed to the same CuSO_4_ concentrations were used as a negative control.
