# Supplementary figures and images for: Membrane Proteomics Analysis of the Candida glabrata Response to 5-Flucytosine: Unveiling the Role and Regulation of the Drug Efflux Transporters CgFlr1 and CgFlr2
Source: Front Microbiol. 2016 Dec 21;7:2045. doi: 10.3389/fmicb.2016.02045 (PMC5174090; doi:10.3389/fmicb.2016.02045)

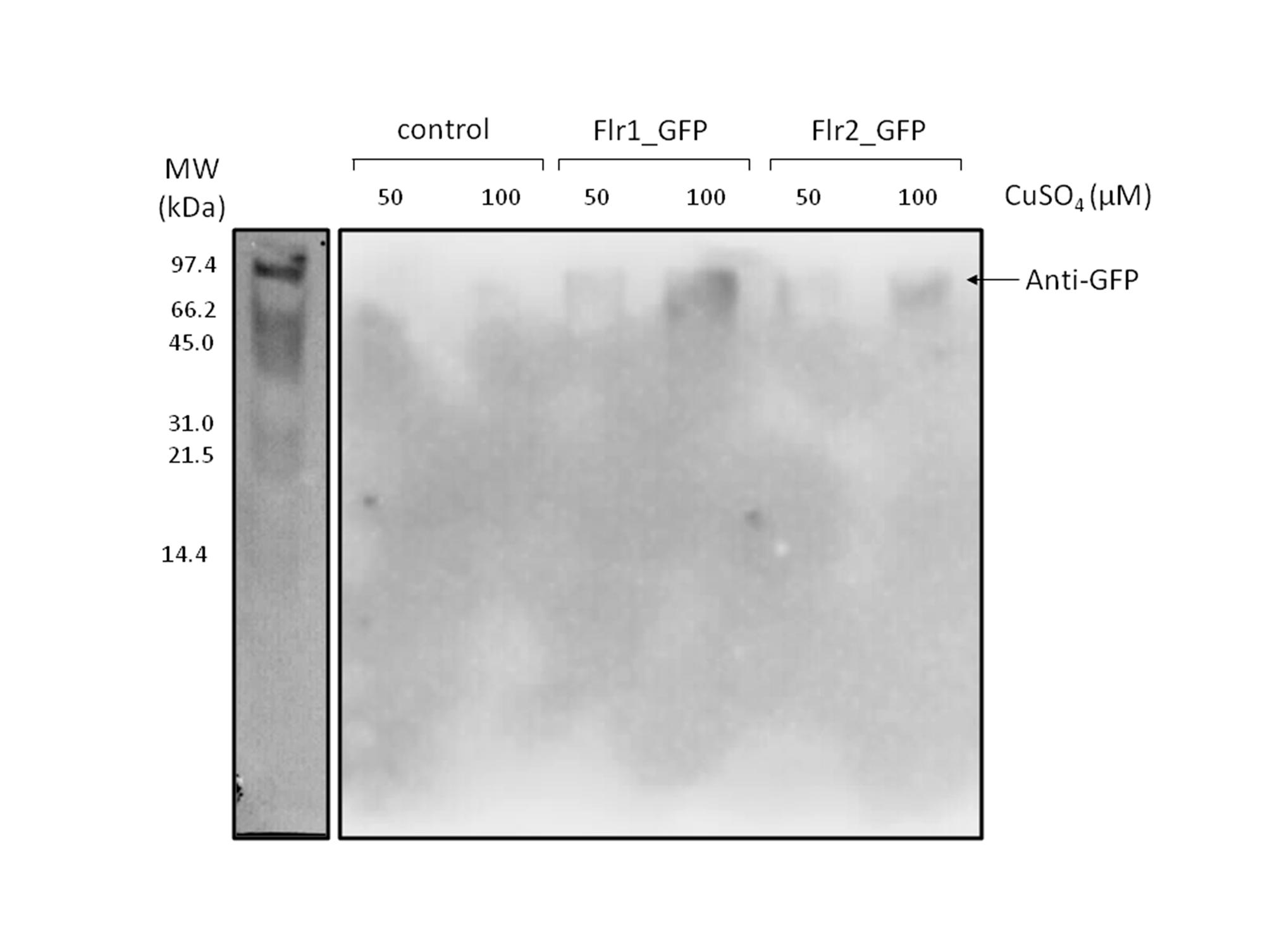

Supplement: Figure S1 — CgFlr1 and CgFlr2 are expressed in C. glabrata cells harboring the pGREG576_MT1_CgFLR1 and the pGREG576_MT1_CgFLR2 plasmids. Comparison of the level of expression of CgFlr1-GFP or CgFlr2-GFP fusion proteins in L5U1 C. glabrata cells upon exposure to the indicated CuSO4 concentrations, based on anti-GFP immuno-detection. Cells harboring the pGREG576 cloning vector (control), exposed to the same CuSO4 concentrations were used as a negative control. [file Image1.TIF]
